# Evaporation-Induced Microphase Separation at the Interface of a Sessile Droplet: Formation of a Regular Lattice Pattern

**DOI:** 10.1021/acs.langmuir.6c04739

**Published:** 2026-09-24

**Authors:** Vahid Nasirimarekani

**Affiliations:** † Max Planck Institute for Dynamics and Self-Organization, Am Fassberg 17, 37077 Göttingen, Germany; ‡ Laboratory of Fluid Physics, Pattern Formation and Biocomplexity, Am Fassberg 17, 37077 Göttingen, Germany

## Abstract

Evaporation alters molecular interactions and can induce phase separation in complex fluids. Whether evaporation can drive localized phase separation at the receding contact line and lead to ordered pattern formation in small liquid volumes remains an open question. Here, we investigate the evaporation-induced microphase separation of a block copolymer (Pluronic F-127) in a salt-containing buffer within an evaporating sessile droplet. We observe that salt-mediated phase separation occurs at the dynamic interface of the droplet, leading to the formation of isolated polymer clusters or highly regular lattice patterns depending on the initial polymer concentration. Our results demonstrate that localized evaporative concentration and salt–polymer interactions at the droplet interface drive salting-out effects and microphase separation into ordered mesophases. This study highlights the utility of evaporating sessile droplets for engineering self-assembled, regular polymeric patterns via interface-mediated microphase separation.

## Graphical abstract

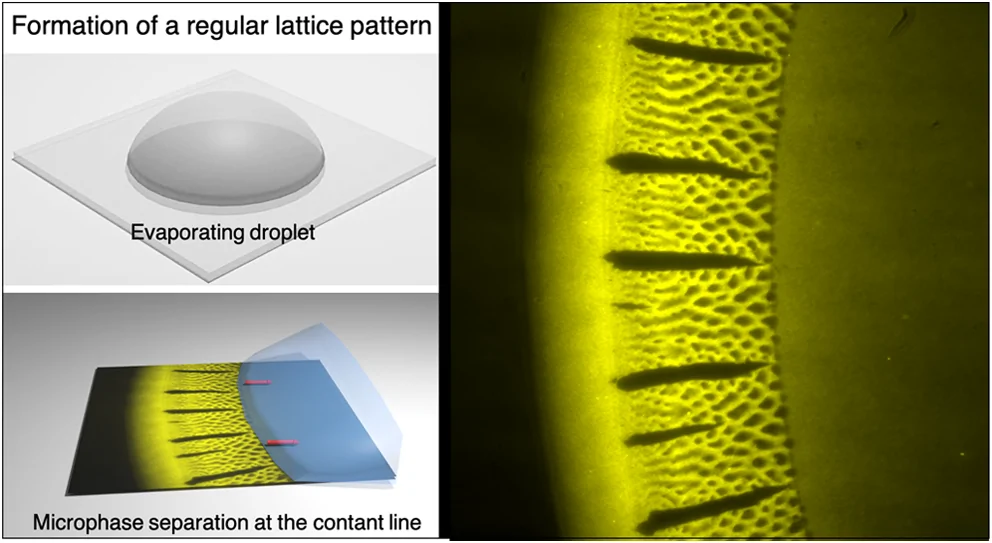

## Introduction

The liquid phase is arguably the most complex state of matter, providing the dynamic medium in which molecular self-assembly and physical organization occur. Its fluidity enables the dissolution and integration of a wide range of solutes, macromolecules, and even solid particles, which can arrange either randomly or into ordered structures. Among the key thermodynamic phenomena occurring in complex fluids, phase separation drives dissolved macromolecules to reorganize into distinct spatial patterns.^1,2^ Depending on molecular architecture and connectivity, such processes range from liquid–liquid phase separation (LLPS) and coacervation to microphase separation, where covalent constraints induce the formation of ordered, periodic mesophases.^3–7^

Confining a liquid to smaller volumes can promote local phase separation by amplifying the influence of concentration gradients and interfacial effects.^5–9^ It also allows the entire volume to be observed, enabling direct visualization of how components organize under nonequilibrium conditions. A sessile droplet, a small volume resting on a solid surface, offers an ideal platform for such studies, as it provides full optical access to the liquid and its contents.^10^ This setup has therefore been widely used to investigate self-assembly and pattern formation under well-controlled environmental conditions.^11–13^

Evaporation is a widespread process that drives gradients in concentration,^13^ temperature,^14^ and surface tension^15,16^ within volatile liquids. These gradients induce precisely the types of thermodynamic shifts that can trigger localized phase transitions. Indeed, evaporation of a volatile solvent promotes the phase separation and self-assembly of dissolved solutes.^17,18^ An evaporating aqueous droplet containing salt and an amphiphilic block copolymer is a particularly relevant example, as localized solvent loss progressively concentrates both the salt and polymer near the receding contact line. This naturally raises the question: what specific structural patterns emerge from the dynamic evaporation of such a multicomponent droplet?

Amphiphilic block copolymers lower the surface tension of an evaporating droplet by migrating to and accumulating at the air–water interface (Figure. [Fig fig1]a).^19,20^ However, salt ions can significantly modulate this interfacial behavior.^21^ Here, we investigate this interplay by studying the pattern formation of an amphiphilic block copolymer at the interface of an evaporating sessile droplet. Specifically, we ask whether the dynamic, shrinking interface can induce localized microphase separation of the polymer chains near the receding contact line (Figure. [Fig fig1]b). We hypothesize that such interfacial microphase separation may give rise to ordered, regular patterns, rather than the classic coffee-ring effect^22,23^ or unstructured isotropic deposition. To test this, we used the nonionic block copolymer Pluronic F-127 (hereafter Pluronic) dissolved in a salt-containing buffer with mono- and multivalent salts at near-physiological pH (∼6.9). Since Pluronic preferentially accumulates at interfaces and concentration gradients intensify near the receding contact line, we focused our visualization on the droplet periphery (Figure 1b, inset).

**Figure 1:**
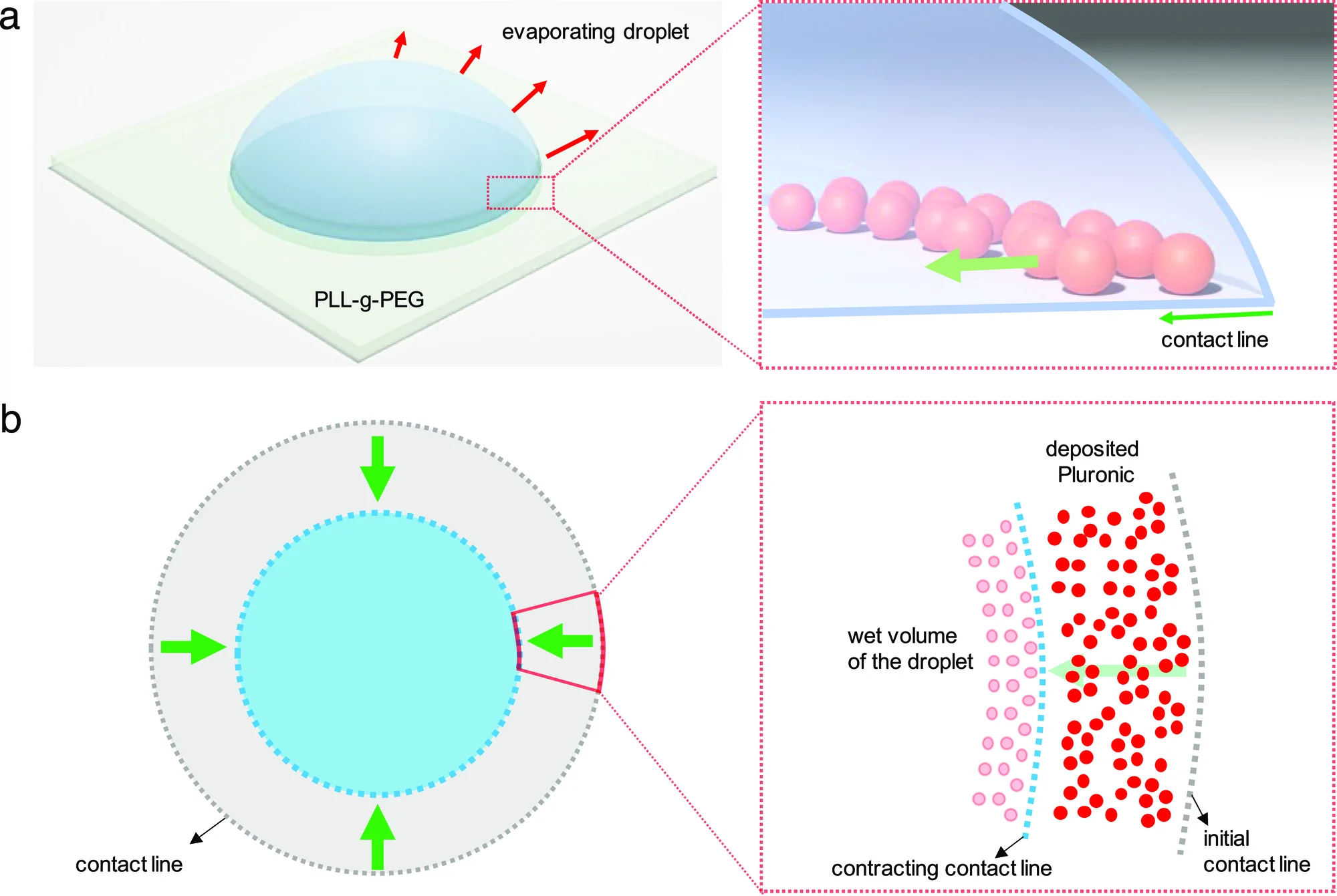
Schematic of the contracting sessile droplet used to study pattern formation of Pluronic polymers at the interface. (a) Overall droplet geometry on a glass substrate functionalized with PLL-*g*-PEG to prevent pinning and nonspecific binding of Pluronic molecules. The inset shows Pluronic molecules at the solid–water interface, where they reduce surface tension. (b) Two-dimensional representation of the receding contact line as the droplet contracts. The gray dashed line indicates the initial contact line, while the blue dashed line represents the contracting contact line. As the droplet recedes, Pluronic molecules deposit on the substrate (shown as red dots in the inset).

In this article, we demonstrate that evaporation-driven salt–polymer interactions at the contact line trigger localized microphase separation, resulting in the self-assembly of a highly regular Pluronic lattice on the substrate. Through a series of controlled experiments that systematically reduce the system’s complexity, we confirm that this periodic patterning arises specifically from the interplay between salt and the block copolymer. We anticipate that these findings offer insights into the self-assembly behavior of synthetic and biological macromolecules with similar block copolymer architectures. More broadly, our work highlights how evaporative contact-line dynamics can be harnessed as a simple, template-free approach for engineering structured functional coatings and mesoscale polymeric patterns.

## Experimental Section

### Materials

#### Pluronic F-127

Pluronic F-127 powder (cell culture suitable) was purchased from Sigma-Aldrich, Germany. Pluronic F-127 is a nonionic amphiphilic triblock copolymer consisting of a central hydrophobic poly­(propylene oxide) (PPO) block flanked by two hydrophilic poly­(ethylene oxide) (PEO) blocks (PEO_100_–PPO_65_–PEO_100_, *M*_*w*_ ≈ 12.6 kDa). Stock solutions were dissolved in M2B buffer, phosphate-buffered saline (PBS), or Milli-Q water and sterilized prior to use.

#### Tracer Particles and Quantum Dots

Qdot 585 ITK carboxyl quantum dots (Thermo Fisher Scientific, Germany) were added to Pluronic solutions at a final concentration of 20 pM to act as nanoscale fluorescent tracers. Carboxylate-modified fluorescent polystyrene microspheres (2 μm diameter, Bangs Laboratories, Inc., USA) were used as microscale tracers for flow profiling and localized entrapment experiments.

#### Substrates and Reagents

High-resolution glass coverslips (24 × 60 mm, Paul Marienfeld GmbH & Co. KG, Germany) served as substrates. Poly­(l-lysine)-*graft*-poly­(ethylene glycol) (PLL-*g*-PEG) was purchased from SuSoS AG, Switzerland. Hellmanex III solution was purchased from Hellma Analytics, Germany. All chemical buffers were prepared using ultrapure Milli-Q water (18.2 MΩ·cm).

### Substrate Preparation and PLL-*g*-PEG Surface Functionalization

Microscope glass slides were cleaned by washing in 100% ethanol for 10 min, rinsing thoroughly with deionized water, sonicating in acetone for 30 min, and reincubating in ethanol for 10 min at room temperature. The slides were subsequently incubated in a 2% (v/v) Hellmanex III solution for 2 h, extensively rinsed with deionized water, and dried under a filtered dry air flow. Cleaned coverslips were activated via oxygen plasma treatment (FEMTO, Diener Electronic, Germany) for 30 s at 0.5 mbar. A solution of 0.1 mg/mL PLL-*g*-PEG in 10 mM HEPES buffer (pH 7.4) was immediately applied to the activated glass surface and incubated for 30 min at room temperature. The functionalization solution was then aspirated, and slides were dried under filtered dry air flow to yield uniform, nonfouling hydrophilic substrates.

### Preparation of Droplet Solutions

M2B buffer (80 mM PIPES, adjusted to pH 6.9 with KOH, 1 mM EGTA, 2 mM MgCl_2_) served as the primary salt-containing buffer. Pluronic F-127 stock solutions at concentrations of 0.5%, 1%, and 3% w/w were prepared by dissolving appropriate masses of polymer powder in M2B buffer under magnetic stirring until fully dissolved. For nanoscale tracer experiments, 1 μL of quantum dot stock solution was added per 100 μL of Pluronic solution (20 pM final concentration). For hydrodynamic flow tracing and network gelation studies, 2 μm fluorescent polystyrene beads were mixed into the Pluronic solutions. Control solutions were prepared identically using either standard phosphate-buffered saline (PBS, pH 7.4) or salt-free Milli-Q water.

### Evaporating Droplet Experiments

All evaporation experiments were conducted under controlled ambient room conditions (22 ± 1 °C, relative humidity 45–50%). A 0.2 μL droplet of the prepared solution was gently deposited onto a freshly passivated PLL-*g*-PEG glass slide mounted on the microscope stage. To protect the evaporating droplet from ambient air currents and ensure isotropic radial drying, a transparent enclosure (10 ×10 × 2 cm) was placed directly over the substrate during image collection.

### Image Acquisition and Quantitative Analysis

#### Image Acquisition

Time-lapse fluorescence and polarized light imaging were performed on an inverted microscope (Olympus IX-71) equipped with objectives ranging from 4× (NA 0.13, Olympus) to 60× oil-immersion (NA 1.35, Olympus). Fluorescence excitation was provided by a metal arc lamp system (Lumen 200, Prior Scientific Instruments, USA). Images were recorded using a CCD camera (CoolSnap HQ2, Photometrics). Image acquisition rates were dynamically adjusted from 1 frame/s during initial evaporation to 5 frames/s during rapid contact-line recession.

#### Image and Hydrodynamic Analysis

Image processing and initial data extraction were performed using ImageJ/Fiji (NIH, USA). Automated particle tracking analysis (PTA) of 2 μm tracer beads was conducted using the TrackMate plugin in Fiji to quantify internal fluid trajectories, measure flow velocities, and confirm the absence of Marangoni recirculation. Contact-line speed and normalized droplet radius over time were extracted by tracking the peripheral interface using automated thresholding. Spatial intensity variations and localized phase accumulation were evaluated using radial gray-value intensity profiling. Custom Python scripts utilizing OpenCV, scikit-image, Matplotlib, and Seaborn were employed for morphological quantification (cluster segmentation, network wall thickness mapping, void area distribution, and lattice orientation analysis).

## Results and Discussion

The evaporation of sessile droplets containing the block copolymer Pluronic F-127 in M2B buffer was visualized using an inverted epi-fluorescence microscope (Figure S1). Quantum dots (QD) with an emission wavelength of 585 nm and an average diameter of 15–20 nm were included as fluorescent tracers to enable high-resolution imaging of the resulting deposition patterns.

We investigated three Pluronic concentrations in the buffer: 0.5%, 1%, and 3% w/w. Given that Pluronic has a critical micelle concentration (CMC) of approximately 0.7% w/w,^24^ these concentrations span both below and above the CMC. Approximately 0.2 μL of each solution was pipetted onto a glass substrate functionalized with poly­(l-lysine)-*graft*-poly­(ethylene glycol) (PLL-*g*-PEG). This brush-like polymer coating prevents nonspecific adsorption of particles and polymers to the surface.^25,26^ The droplets were allowed to evaporate at room temperature while time-lapse images were acquired at the droplet periphery to capture the deposition of Pluronic patterns on the substrate (Figure S1).

### Initial Polymer Concentration Dictates Contact-Line Patterning

Systematic imaging reveals that the initial polymer concentration determines the final deposition pattern. We observe three distinct morphologies as a function of Pluronic concentration: (i) disordered aggregate clusters at 0.5% w/w, (ii) a regular lattice pattern at 1% w/w, and (iii) a continuous sheet with radially aligned defects at 3% w/w (Figure 2).

**Figure 2:**
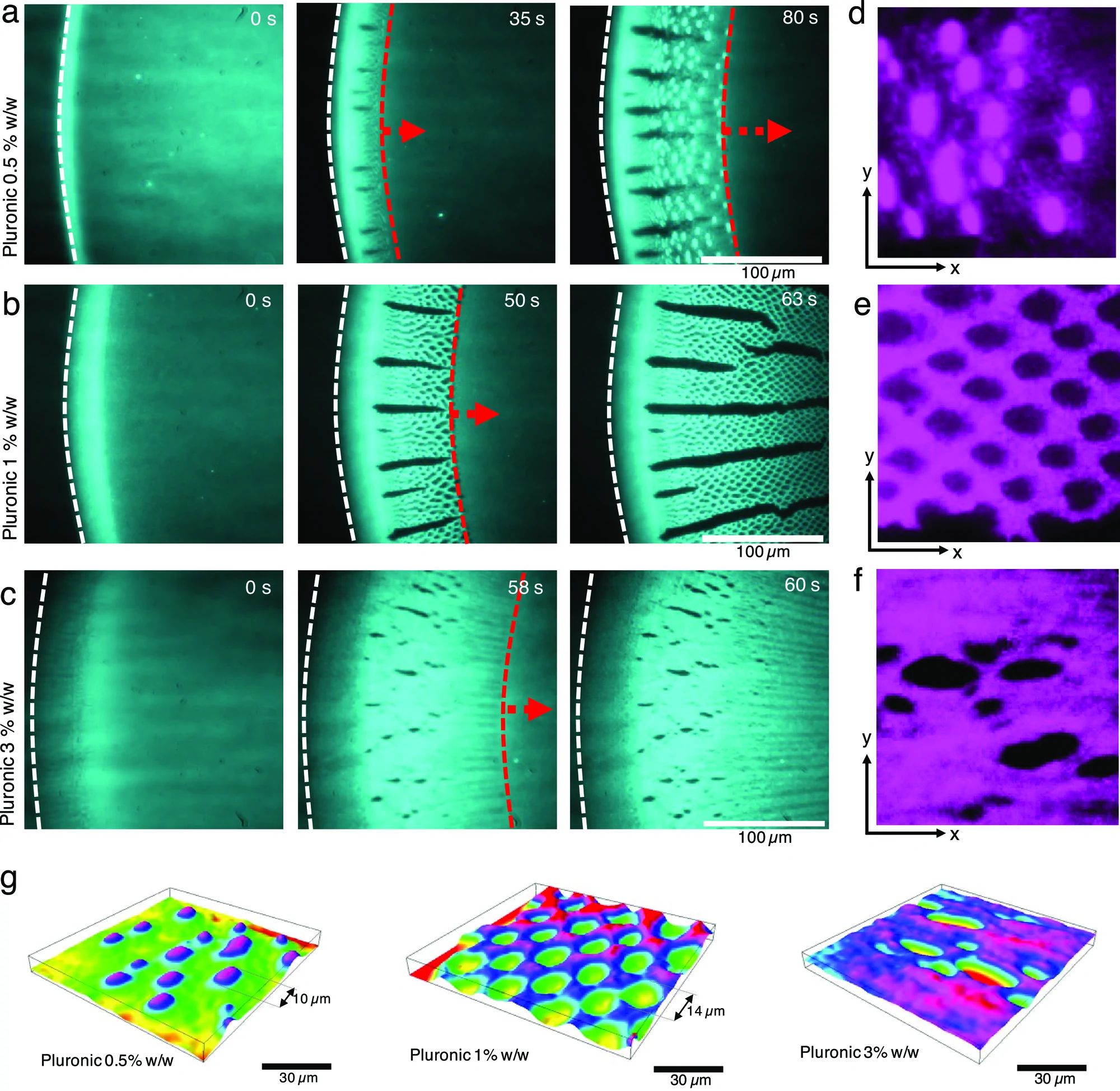
Effect of Pluronic concentration on pattern formation at the solid–water–air interface of the evaporating droplet. (a–c) Time-lapse images of droplets containing 0.5%, 1%, and 3% w/w Pluronic, respectively. Increasing the Pluronic concentration drives a transition from clustered aggregates (0.5%) to a regular lattice pattern (1%) and finally to continuous sheets with radial finger-like defects (3%). (d–f) Enlarged views of the corresponding patterns. (g) Three-dimensional surface topology plots based on fluorescence intensity for the patterns shown in (d–f).

At 0.5% Pluronic, dense clusters deposit at the interface, surrounded by a thin layer (Figure 2a,d). These clusters form at the air–droplet interface (contact line) and are irregularly distributed along it (Figure 2a, 80 s; red dashed line indicates the contracting contact line and white dashed line represents the initial contact line of the droplet). The cluster density decreases radially toward the droplet center, and radial striations are observed perpendicular to the contact line.

For the 1% droplet, a polymeric sheet with a distinct lattice pattern and more pronounced radial striations is observed (Figure 2b,e). The lattice exhibits regular, linearly aligned features covering a large fraction of the droplet area. At 3% Pluronic, the pattern transitions to a continuous sheet with less distinct striations that appear as finger-like radial defects (Figure 2c,f). Three-dimensional surface topology plots, generated from fluorescence intensity profiles, corroborate these observations (Figure 2g).

Importantly, these self-assembly processes occur localized at the three-phase contact line (solid–water–air interface, Figure 1), where polymer material deposits progressively onto the substrate as the contact line recedes (Videos S1 and S2). Fluorescence visualization across all experiments was enabled by incorporating quantum dots as passive tracers. Control experiments performed in the complete absence of quantum dots yielded identical deposition morphologies (Figure S2), confirming that these tracer nanoparticles do not influence the phase behavior or pattern formation.

These experiments demonstrate that the initial Pluronic concentration significantly influences the final deposition pattern. Notably, the emergence of a regular lattice pattern at intermediate concentration suggests that precise tuning of the initial polymer concentration can yield highly ordered structures. While previous studies have reported that initial polymer concentration affects the final pattern,^27–29^ our results reveal a more complex transition, from randomly distributed clusters to an ordered lattice, as a function of the initial concentration.

### Quantitative Characterization of Cluster Assembly and Lattice Symmetry

To quantify the growth dynamics of the aggregate clusters formed at 0.5% Pluronic, we performed time-resolved image segmentation on time-lapse sequences (Figure 3a–b). This analysis reveals a pronounced, monotonic increase in both the total cluster count and the net surface area coverage as evaporation progresses. During this process, the clusters are pulled strictly inward along the radial direction following the receding contact line, with no detectable lateral motion. This steady growth and directional translation reflect continuous local accumulation and aggregation of Pluronic driven by evaporative flux at the peripheral interface.

**Figure 3:**
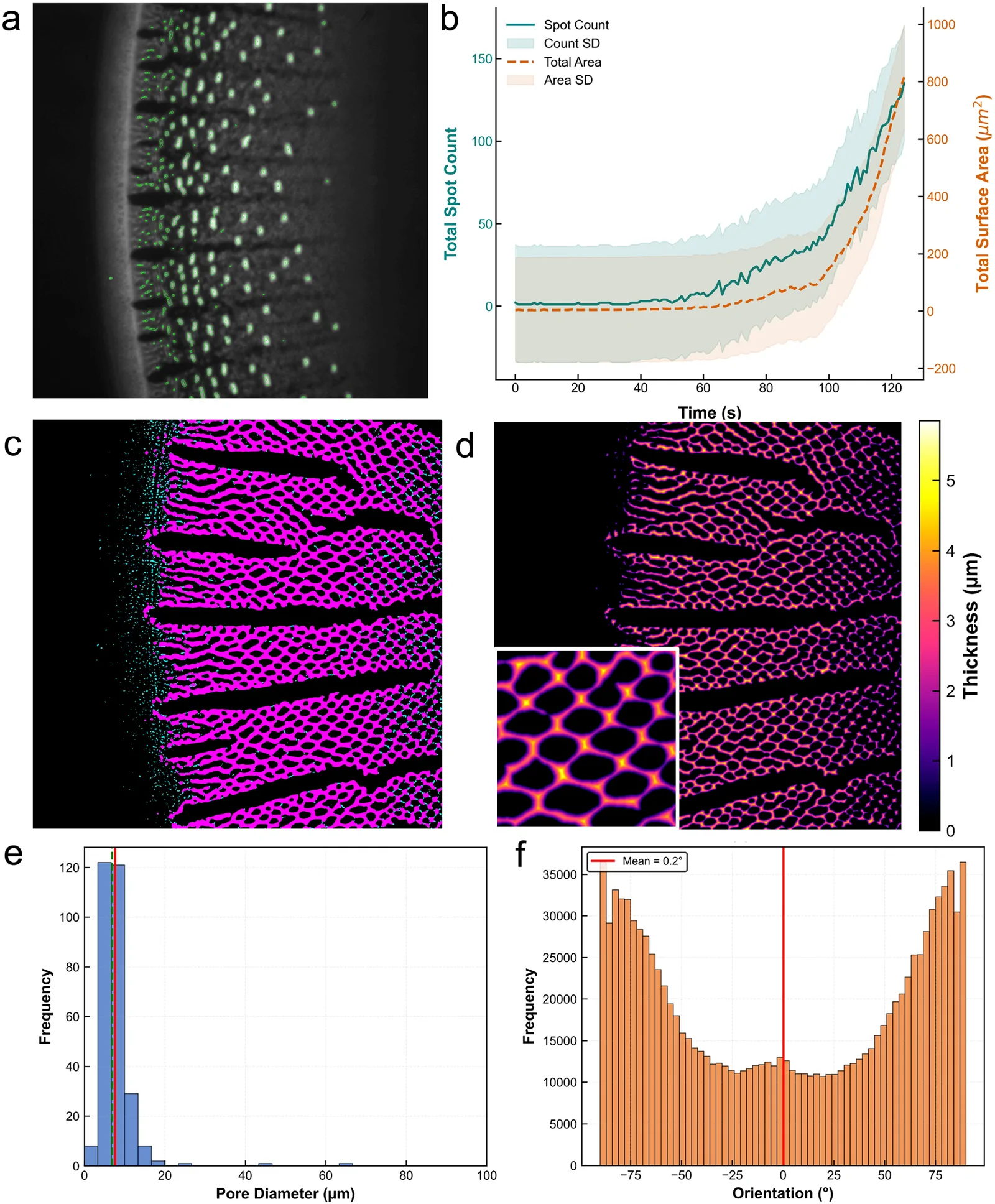
Quantification of microphase separation and pattern morphology at the interface of the evaporating droplet. (a, b) Representative segmentation of clusters formed at the interface over time, with corresponding plots showing the total cluster count and surface area coverage during evaporation. (c) Segmentation of the lattice pattern for the 1% w/w droplet (highlighted in pink). (d) Color-coded thickness map of the lattice pattern in (c), showing higher accumulation at line intersections. (e) Size distribution of lattice voids, displaying a mean value of approximately 7.6 μm. (f) Orientation distribution of the lattice pattern, confirming the high structural symmetry of the voids.

For the 1% droplet, we further characterized the spatial organization of the microphase-separated lattice using morphological segmentation (Figure 3c). Mapping the local network thickness reveals that the maximum thickness consistently occurs at the lattice line intersections (Figure 3d), indicating that these nodes serve as primary accumulation points during the self-assembly process. Morphological analysis of the void size distribution yields a narrow distribution centered at a mean characteristic length scale of approximately 7.6 μm (Figure 3e). Furthermore, the lattice orientation distribution peaks sharply near zero (Figure 3f), quantitatively confirming the isotropic symmetry, spatial uniformity, and regular alignment of the interconnected voids.

### Interfacial Microphase Separation and Lattice Patterning Dynamics

The distinct deposition morphologies observed at the contact line suggest a concentration-dependent phase separation driven by localized evaporation. As solvent evaporates, progressive concentration of the amphiphilic block copolymer near the peripheral interface triggers local phase separation. At 0.5% Pluronic, this manifests initially as discrete clusters along the receding contact line. Time-lapse microscopy shows that these small clusters continuously coalesce into larger aggregates as the contracting interface pushes them together (Figure 4a,c,d). These clusters are dragged strictly inward along the radial path of the receding contact line, leaving behind a characteristic footprint (Figure 4a, yellow arrow), while nonclustered chains deposit as a thin background film (Figure 4b).

**Figure 4:**
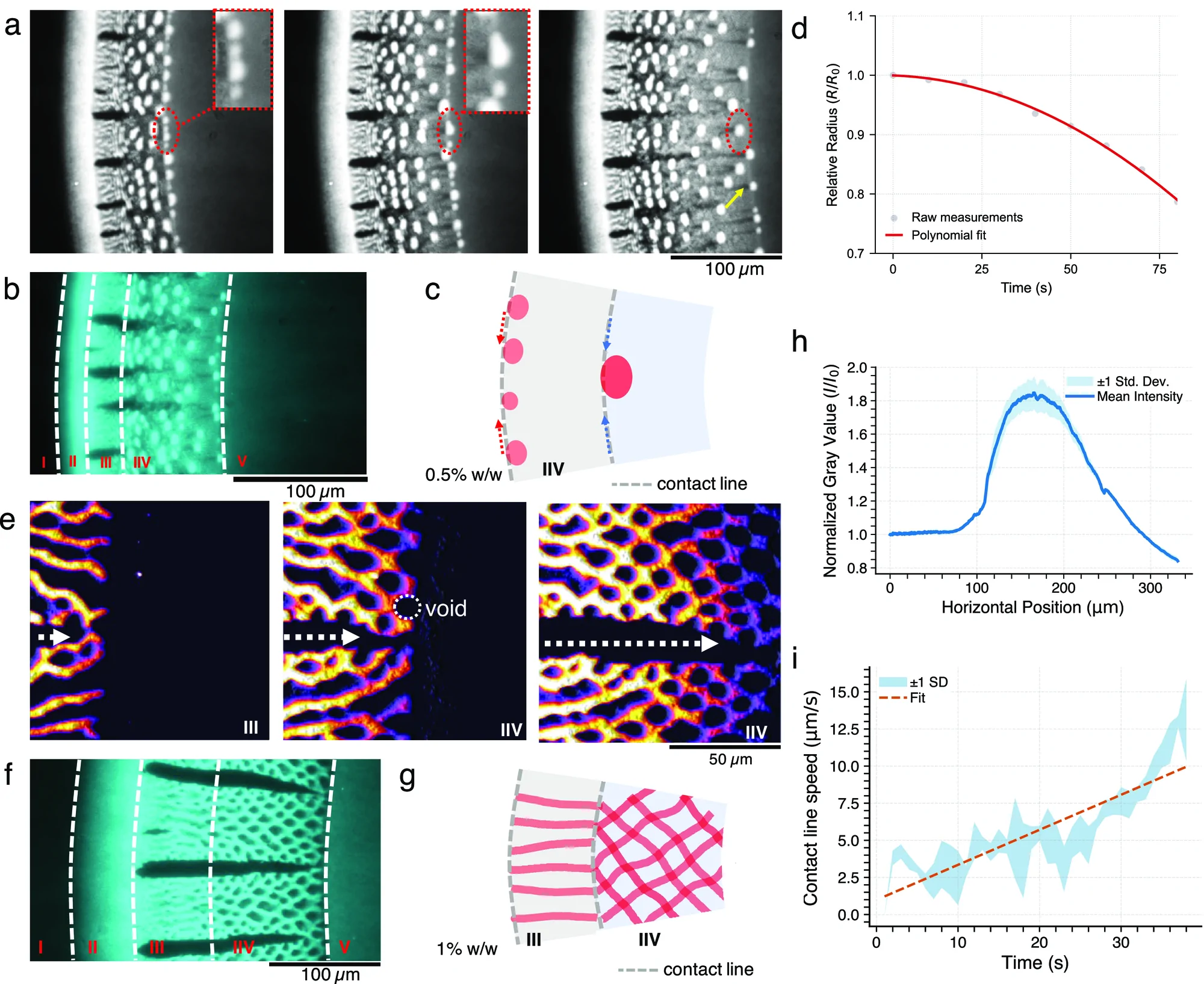
Detailed view of Pluronic pattern formation at the dynamic droplet interface. (a) Experimental observation of polymer cluster formation and coalescence driven by contact-line recession. (b) Spatial pattern variation for a 0.5% w/w Pluronic droplet across radial regions. (c) Schematic of polymer coacervate merging into larger clusters. (d) Normalized droplet radius (*R*/*R*_0_) over time during cluster formation (0.5% w/w). (e) Time-series showing microphase separation near the contact line, transitioning from finger-like instabilities to a regular lattice pattern with interfacial voids. (f) Spatial pattern variation for a 1% w/w Pluronic droplet. (g) Schematic illustrating the transition to a lattice pattern across distinct structural zones. (h) Radial gray-value profile showing microphase separation at maximum local polymer concentration. (i) Contact-line velocity during lattice formation showing a linear increase over time.

At an initial concentration of 1% Pluronic, the system shifts from discrete, isolated aggregates to an ordered, interconnected lattice network (Figure 4e–g). This architectural transition is governed by interfacial microphase separation, wherein local evaporative water loss elevates the polymer concentration past the critical concentration at the contact line. Radial intensity profiles confirm that microphase separation initiates specifically at the region of maximum local Pluronic accumulation near the periphery (Figure 4h). As local solubility limits are exceeded, thermodynamic microphase segregation between the hydrated poly­(ethylene oxide) (PEO) and hydrophobic poly­(propylene oxide) (PPO) blocks drives the spontaneous self-assembly of closely packed mesodomains rather than macroscale precipitation.^30,31^

To understand the hydrodynamic feed underlying these concentration-dependent phenomena, we incorporated 2 μm fluorescent polystyrene beads as flow tracers within the evaporating droplet (Figure S3 and Video S4). Particle tracking analysis reveals a continuous, unidirectional outward flow directed toward the receding contact line throughout the evaporation process, with no circulating Marangoni eddies or inward backflows detected. This observation confirms that the transport of polymer chains and micelles to the peripheral interface is overwhelmingly governed by classic capillary-driven flow, which continuously feeds the local accumulation zone where microphase separation takes place.

Having established that outward capillary flow continuously delivers solute to the droplet edge, we propose a mechanistic hypothesis for the formation of the ordered lattice based on the coupled dynamics of phase separation and contact-line motion (Figure 4g). As the contact line recedes, evaporative accumulation first drives the formation of parallel, finger-like radial phase domains (Region III, Figure 4e,g). As evaporation progresses, the contact-line velocity accelerates linearly over time (Figure 4i), while the total droplet radius decreases (Figure 4d). We hypothesize that as the contact line accelerates and the interface contracts, the continuous influx of polymer destabilizes the elongated finger-like structures. This dynamic instability transforms the linear fingers into an interconnected network of polymer-rich lines surrounding circular voids (Region IV, Figure 4e,g; white arrows indicate radial defect lines). Nevertheless, we acknowledge that further theoretical and high-resolution rheological studies are needed to fully model the instabilities governing this pattern genesis.

Reflecting this physical transformation, the structural integrity of the resulting microphase-separated network suggests thin-film gel-like properties. At 1% w/w (above the critical micelle concentration), the solution contains a mixture of free unimers and hydrated micelles, which significantly alters the local viscoelasticity near the interface.^32,33^ This gel-like state is further supported by tracer bead behavior (Figure S4), where embedded beads become immobilized and trapped within the polymer matrix rather than freely diffusing, reflecting a localized transition toward a gel-like continuous sheet.

Finally, while local polymer concentration and contact-line dynamics dictate these interfacial instabilities, we hypothesize that the presence of salt modulates block copolymer solubility and shifts the microphase separation threshold. The influence of salt on this pattern formation is addressed in the following section.

### Salting-Out Effect Modulates Interfacial Microphase Separation

To determine the necessity of electrolytes in driving interfacial phase separation and lattice formation, we performed control experiments in Milli-Q water (in the complete absence of salt) while maintaining identical polymer concentrations (1% and 3% w/w) and drying conditions.

In the absence of salt, no microphase separation or ordered lattice patterns were observed (Figure 5). Instead, the final deposition morphology differed markedly from electrolyte-containing droplets. At 1% w/w Pluronic in water, although five distinct radial zones analogous to those in Figure 4g could still be identified, the material deposited as a featureless film lacking regular microscopic patterns or radial defect lines (Figure. [Fig fig5]a). Increasing the concentration to 3% w/w yielded a uniform, continuous sheet with no visible radial fingering (Figure. [Fig fig5]b). Interestingly, intermediate control experiments using standard phosphate-buffered saline (PBS) exhibited finger-like radial instabilities near the contact line, yet failed to transition into the fully connected, highly symmetric lattice network observed in our multisalt buffer system (Figure S5).

**Figure 5:**
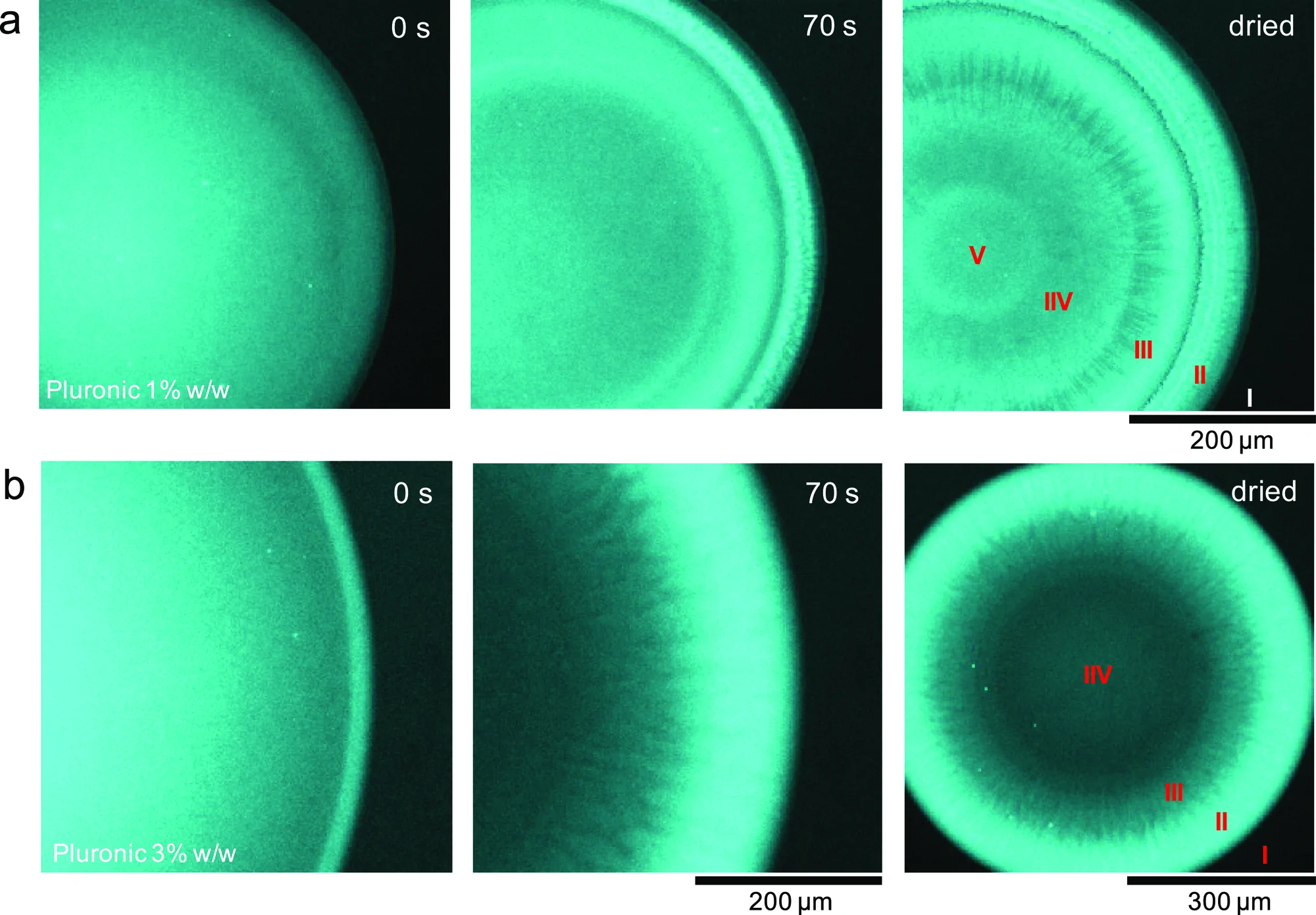
Pattern formation of Pluronic block copolymer in pure water (absence of salt) within an evaporating droplet. (a, b) Time-lapse images of droplets containing 1% and 3% w/w Pluronic, respectively. In the complete absence of salt, no microphase separation, regular lattice assembly, or radial defect networks occur.

These comparative observations demonstrate that electrolyte presence is a strict prerequisite for driving interfacial microphase separation in this system. Dissolved salts lower the solubility of nonionic block copolymers by screening polymer–water hydrogen bonding and competing for hydration water, a classic salting-out effect.^34–36^ By reducing the effective quality of the solvent, electrolytes enhance unfavorable interactions between water and the hydrophobic PPO blocks, thereby shifting the binodal phase boundary and promoting local phase segregation at lower polymer thresholds.^37,38^

In addition to shifting phase boundaries, electrolyte accumulation in evaporating droplets fundamentally alters mass transport and interfacial dynamics. As demonstrated in recent studies of salt-containing evaporating droplets, localized solute concentration promotes distinct crystallization patterns and mechanical instabilities at the contact line.^39,40^ In our polymer–salt system, the presence of specific buffer salts similarly modulates the viscoelastic properties and gelation kinetics of Pluronic solutions near the interface. The complete absence of radial defect lines and void pinching in salt-free controls further suggests that electrolyte-induced changes in thin-film mechanics are essential for stabilizing the microphase-separated network geometry.

Additionally, because the physiological buffer used here contains a mixture of monovalent and polyvalent ions, the difference in pattern development between pure water, PBS, and our complete buffer demonstrates that salt formulation directly dictates domain stability and network architecture.^41^ We hypothesize that systematically varying ion valency and ionic strength via the Hofmeister series could serve as a precise route to tune lattice void size and structural periodicity, offering a promising direction for future studies on self-assembled patterning.

## Conclusions

We have demonstrated that the evaporation of a sessile droplet containing the nonionic block copolymer Pluronic F-127 in an electrolyte buffer induces interfacial microphase separation at the receding contact line, yielding distinct, concentration-dependent deposition patterns. At low concentration (0.5% w/w), phase separation produces discrete, disordered polymer clusters along the interface. At 1% w/w, the system self-assembles into a highly ordered lattice network characterized by a uniform void size of approximately 7.6 μm and high structural symmetry, with preferential polymer accumulation at the lattice intersections. At high concentration (3% w/w), this architecture transitions into a continuous gel-like sheet containing radial defect lines.

Control experiments in pure water confirmed that the presence of salt is essential for triggering this microphase separation, which we attribute to salting-out effects that lower polymer solubility and shift the phase boundary near the concentrating interface. Electrolyte interactions also alter the local viscoelasticity of the evaporating thin film, contributing to the structural integrity and defect dynamics of the final patterns.

Our findings establish evaporation-induced interfacial microphase separation in sessile droplets as a simple, versatile route for generating ordered surface microstructures. The ability to dictate pattern morphology through initial polymer concentration, combined with parameters such as salt formulation, humidity, and temperature, offers a promising direction for future research on template-free surface patterning and the controlled fabrication of microstructured polymeric materials.

## Supplementary Material











## Data Availability

The data that support the findings of this study are available within the article and its Supporting Information. Source raw data are available from the corresponding authors upon reasonable request.

## Notes

Additional experimental details, including the experimental setup schematic, polarized light microscopy control data, particle-tracking capillary flow analysis, mechanical property characterization using polystyrene beads, deposition dynamics in phosphate-buffered saline (PBS) (PDF)
Time-lapse fluorescence microscopy of an evaporating sessile droplet containing 0.5% w/w Pluronic F-127 in M2B buffer, showing the formation and merging of coacervate clusters at the receding contact line (Video S1) (AVI)
Time-lapse fluorescence microscopy of an evaporating sessile droplet containing 1% w/w Pluronic F-127 in M2B buffer, showing the emergence of the regular lattice pattern and radial rips at the receding contact line (Video S2) (AVI)
Time-lapse microscopy of the lattice pattern formation in a 1% w/w Pluronic droplet, highlighting the propagation of radial rips through the lattice sheet and the resulting crack-like defects that do not disrupt the overall lattice structure (Video S3) (AVI)
Side-by-side view of the evaporating droplet. The left panel shows polarized light microscopy revealing the lattice pattern, and the right panel displays fluorescent imaging of the 2 μm beads used to track the internal flow of the droplet throughout the evaporation (Video S4) (AVI)

## References

[1] Cahn J. W. (1965). Phase Separation
by Spinodal Decomposition in Isotropic Systems. J. Chem. Phys..

[2] Jones, R. Soft Condensed Matter, Oxford Master Series in Physics; OUP: Oxford, 2002.

[3] Sing C. E., Perry S. L. (2020). Recent progress
in the science of complex coacervation. Soft
Matter.

[4] Quiroz F. G., Fiore V. F., Levorse J., Polak L., Wong E., Pasolli H. A., Fuchs E. (2020). Liquid-liquid
phase separation drives
skin barrier formation. Science.

[5] Rai R., Gopu M., Parameswaran S. P., Adhyapak T. C., Mampallil D. (2024). Dynamics of
phase-separated microdroplets near the contact line of evaporating
all-aqueous drops. Soft Matter.

[6] May A., Hartmann J., Hardt S. (2022). Phase separation
in evaporating all-aqueous
sessile drops. Soft Matter.

[7] Li Y., Lv P., Diddens C., Lohse D. (2022). Physiochemical hydrodynamics of the
phase segregation in an evaporating binary microdroplet. J. Fluid Mech..

[8] Villois A., Palmiero U. C., Mathur P., Perone G., Schneider T., Li L., Salvalaglio M., DeMello A., Stavrakis S., Arosio P. (2022). Droplet Microfluidics
for the Label-Free Extraction
of Complete Phase Diagrams and Kinetics of Liquid-Liquid Phase Separation
in Finite Volumes. Small.

[9] Shimizu S., Matubayasi N. (2021). Phase stability condition and liquid-liquid phase separation
under mesoscale confinement. Phys. A.

[10] Garcia-Cordero J. L., Fan Z. H. (2017). Sessile droplets
for chemical and biological assays. Lab Chip.

[11] Weber E. H. (1855). Mikroskopische
Beobachtungen sehr gesetzmässiger Bewegungen, welche die Bildung
von Niederschlägen harziger Körper aus Weingeist begleiten. Ann. Phys..

[12] Guo W., Kinghorn A. B., Zhang Y., Li Q., Poonam A. D., Tanner J. A., Shum H. C. (2021). Non-associative phase separation
in an evaporating droplet as a model for prebiotic compartmentalization. Nat. Commun..

[13] Bell A. K., Kind J., Hartmann M., Kresse B., Höfler M. V., Straub B. B., Auernhammer G. K., Vogel M., Thiele C. M., Stark R. W. (2022). Concentration gradients
in evaporating binary droplets
probed by spatially resolved Raman and NMR spectroscopy. Proc. Natl. Acad. Sci. U.S.A..

[14] Fang G., Ward C. (1999). Temperature measured close to the interface of an evaporating liquid. Phys. Rev. E.

[15] Shereshefsky J. L., Steckler S. (1936). A study of the evaporation of small drops and of the
relationship between surface tension and curvature. J. Chem. Phys..

[16] Jahangir R., Kim Y., Nasirimarekani V. (2025). Self-Propulsion and a Push-Pull Mechanism
in Sessile Droplets. Langmuir.

[17] Lohse D., Zhang X. (2020). Physicochemical hydrodynamics
of droplets out of equilibrium. Nat. Rev. Phys..

[18] Othman A. M., Poulos A. S., Torres O., Routh A. F. (2023). Liquid-Liquid Phase
Separation Induced by Vapor Transfer in Evaporative Binary Sessile
Droplets. Langmuir.

[19] Marin A., Liepelt R., Rossi M., Kähler C. J. (2016). Surfactant-driven
flow transitions in evaporating droplets. Soft
Matter.

[20] Shao X., Duan F., Hou Y., Zhong X. (2020). Role of surfactant
in controlling the deposition pattern of a particle-laden droplet:
Fundamentals and strategies. Adv. Colloid Interface
Sci..

[21] Nguyen C. V., Peng M., Duignan T. T., Nguyen A. V. (2022). Salting-up of surfactants
at the surface of saline water as detected by tensiometry and SFG
and supported by molecular dynamics simulation. J. Phys. Chem. B.

[22] Deegan R. D., Bakajin O., Dupont T. F., Huber G., Nagel S. R., Witten T. A. (1997). Capillary flow as the cause of ring
stains from dried
liquid drops. Nature.

[23] Yunker P. J., Still T., Lohr M. A., Yodh A. (2011). Suppression of the
coffee-ring effect by shape-dependent capillary interactions. Nature.

[24] Alexandridis P., Holzwarth J. F., Hatton T. A. (1994). Micellization of poly (ethylene oxide)-poly
(propylene oxide)-poly (ethylene oxide) triblock copolymers in aqueous
solutions: thermodynamics of copolymer association. Macromolecules.

[25] Feng C., Huang X. (2018). Polymer brushes: efficient synthesis and applications. Acc. Chem. Res..

[26] Benetti E. M., Spencer N. D. (2019). Using polymers to impart lubricity
and biopassivity
to surfaces: Are these properties linked?. Helv.
Chim. Acta.

[27] Bormashenko E., Pogreb R., Musin A., Stanevsky O., Bormashenko Y., Whyman G., Gendelman O., Barkay Z. (2006). Self-assembly in evaporated polymer solutions: Influence
of the solution concentration. J. Colloid Interface
Sci..

[28] Tsige M., Grest G. S. (2005). Solvent evaporation and interdiffusion in polymer films. J. Phys.: Condens. Matter.

[29] Schaefer C., van der Schoot P., Michels J. J. (2015). Structuring of polymer
solutions
upon solvent evaporation. Phys. Rev. E.

[30] Teran A. A., Balsara N. P. (2014). Thermodynamics of
Block Copolymers with and without
Salt. J. Phys. Chem. B.

[31] Thelen J. L., Teran A. A., Wang X., Garetz B. A., Nakamura I., Wang Z.-G., Balsara N. P. (2014). Phase behavior
of a block copolymer/salt
mixture through the order-to-disorder transition. Macromolecules.

[32] Xiang W., Zhu Z., Song X., Zhong C., Wang C., Ma Y. (2016). Concentration-induced
structural transition of block polymer self-assemblies on a nanoparticle
surface: computer simulation. RSC Adv..

[33] Nasirimarekani V., Strübing T., Vilfan A., Guido I. (2021). Tuning the properties
of active microtubule networks by depletion forces. Langmuir.

[34] Endo S., Pfennigsdorff A., Goss K.-U. (2012). Salting-out effect
in aqueous NaCl
solutions: trends with size and polarity of solute molecules. Environ. Sci. Technol..

[35] Grover P. K., Ryall R. L. (2005). Critical appraisal of salting-out and its implications
for chemical and biological sciences. Chem.
Rev..

[36] Hey M. J., Jackson D. P., Yan H. (2005). The salting-out
effect and phase
separation in aqueous solutions of electrolytes and poly (ethylene
glycol). Polymer.

[37] Imae T., Sasaki M., Abe A., Ikeda S. (1988). Liquid-liquid phase
separation in dilute aqueous solutions of surfactants: the effect
of added salt. Langmuir.

[38] Aseyev, V. ; Tenhu, H. ; Winnik, F. M. Non-Ionic Thermoresponsive Polymers in Water. In Self Organized Nanostructures of Amphiphilic Block Copolymers II, Advances in Polymer Science; Springer, 2011; Vol. 242, pp 29–89 10.1007/12_2010_57.

[39] Basu N., Mukherjee R. (2020). Evaporative
Drying of Sodium Chloride Solution Droplet
on a Thermally Controlled Substrate. J. Phys.
Chem. B.

[40] Basu N., Mukherjee R. (2018). Morphology modulation in evaporative drying mediated
crystallization of sodium chloride solution droplet with surfactant. Soft Matter.

[41] Liu T., Xie Y., Liu L.-Z., Chu B. (2000). Salt-Induced Microphase Separation
and Crystallization in Salt- Polymer Complex Systems. Langmuir.

