# Supplementary material for: Evaporation-Induced Microphase Separation at the Interface of a Sessile Droplet: Formation of a Regular Lattice Pattern
Source: Langmuir. 2026 Sep 24;42(39):29492–500. doi: 10.1021/acs.langmuir.6c04739 (PMC13648271; doi:10.1021/acs.langmuir.6c04739)
Supplement: Supplementary file 1 [file la6c04739_si_001.pdf]

Supporting Information

Evaporation-induced microphase separation at the interface of a sessile droplet: formation of a regular lattice pattern

Vahid Nasirimarekani[1,2,*]

[1]Max Planck Institute for Dynamics and Self-Organization, Am Fassberg 17, 37077 Göttingen, Germany

[2]Laboratory of Fluid Physics, Pattern Formation and Biocomplexity, Am Fassberg 17, 37077 Göttingen, Germany

[*]Corresponding author: vahid.nasirimarekani@ds.mpg.de

Pages: 8

Supplementary Figures: Figures S1 to S5

Supplementary Videos: Videos S1 to S4

Experimental setup

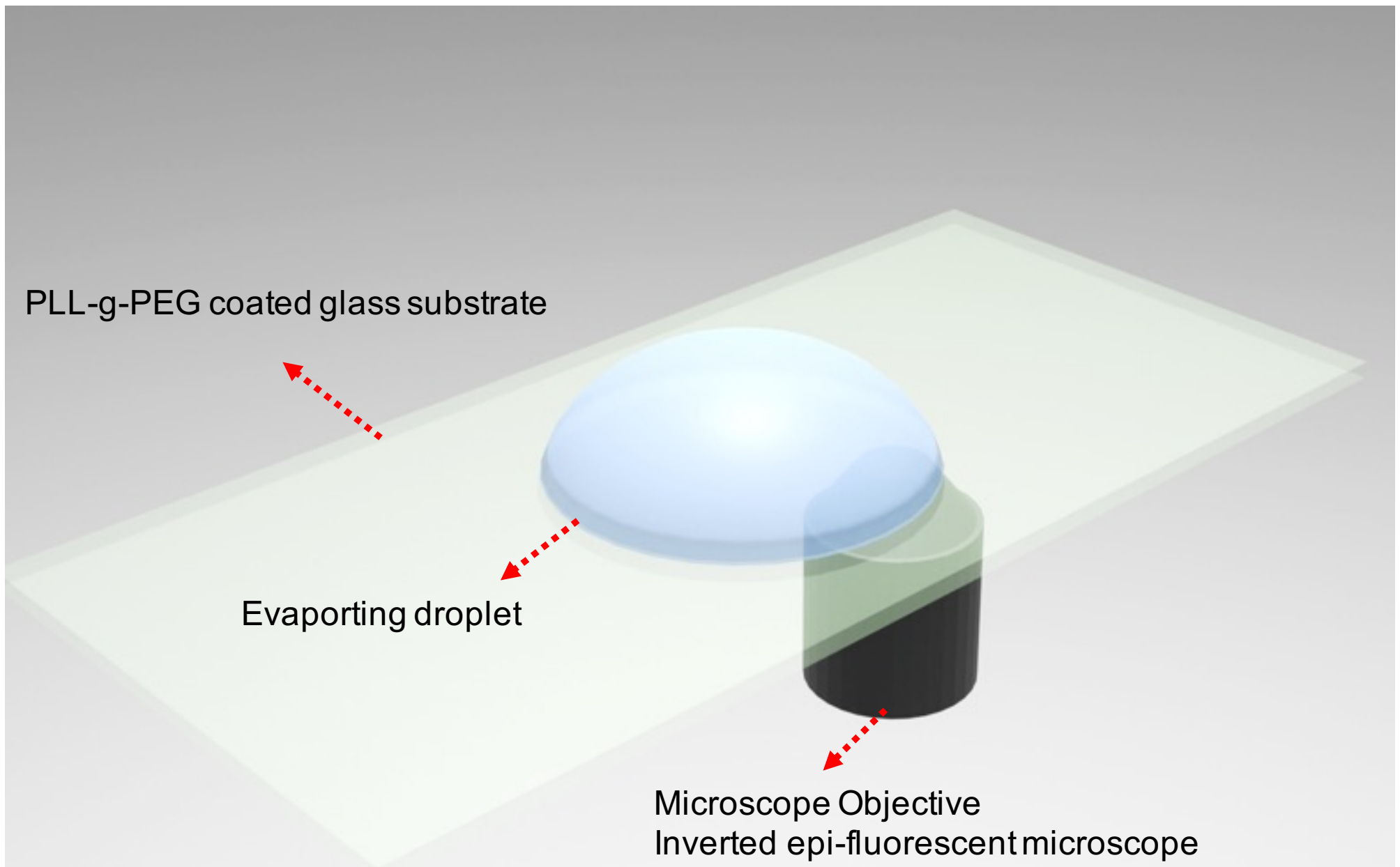

Figure S1: Three-dimensional schematic of the experimental setup. The inverted epifluorescence microscope configuration enables real-time visualization of the droplet from below, allowing observation of the receding contact line and resulting deposition patterns on the PLL-g-PEG-coated glass substrate.

Lattice pattern formation without tracer particles

To determine whether the quantum dots used as fluorescent tracers influence the final pattern formation, we performed control experiments in the absence of quantum dots. The resulting patterns were imaged using polarized light microscopy, which provides contrast based on the birefringent properties of the polymeric material without the need for fluorescent labeling.

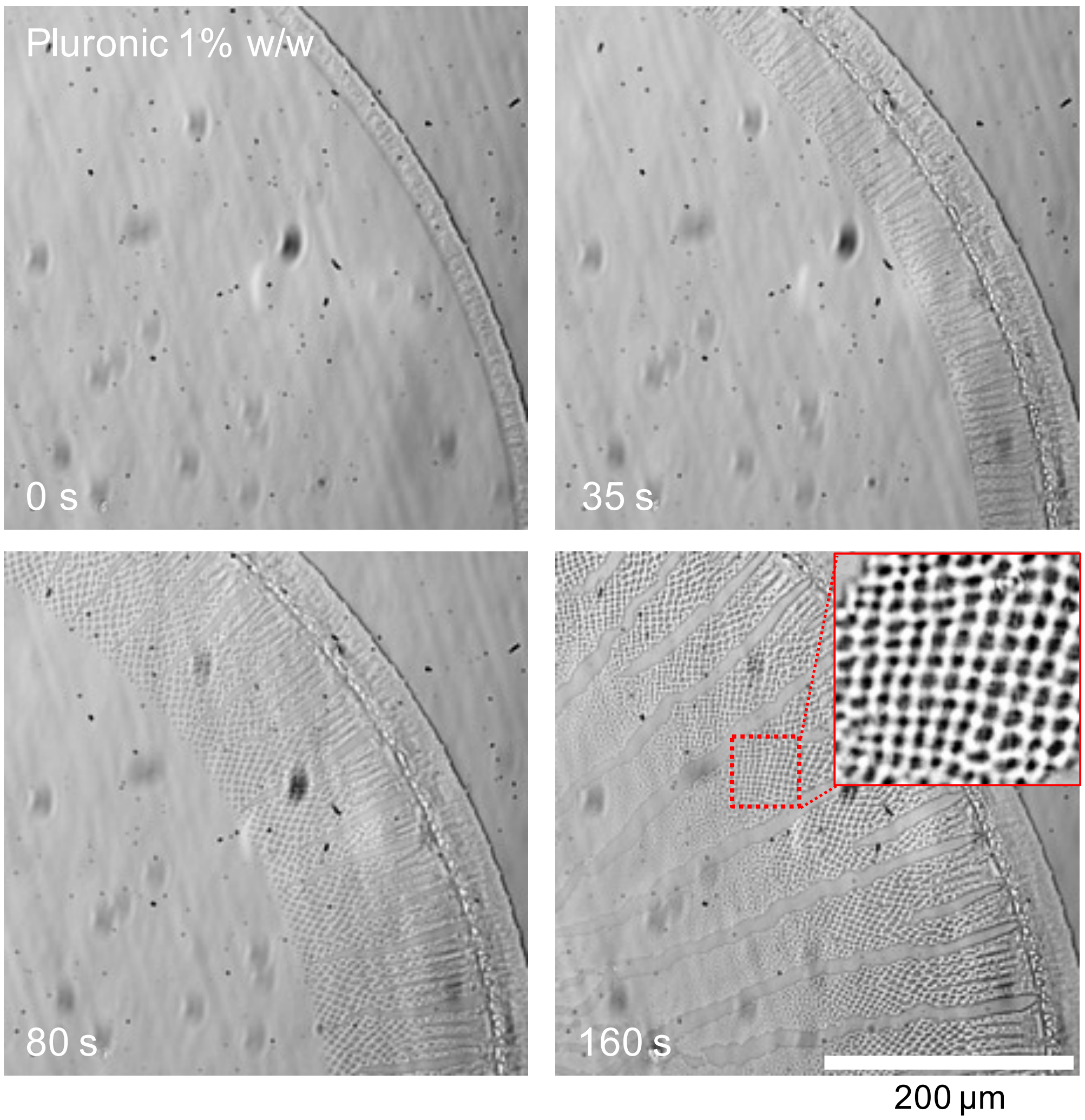

Figure S2: Polarized light microscopy of the lattice pattern formed without quantum dots. The inset provides a magnified view. Comparison with fluorescence images (Figure. 2) reveals no discernible differences, confirming that quantum dots do not interfere with pattern formation.

Outward capillary flow

The analysis of the particle tracking data reveals distinct transport dynamics within the system. First, the velocity vector field demonstrates a consistent outward capillary flow radiating from the droplet center toward the contact line (Figure. 3a,b). Second, the mean squared displacement (MSD) analysis yields a power-law exponent of $\alpha = 1.93$ ($R^2 = 1.00$), indicating directed, ballistic transport rather than random thermal diffusion (Figure. 3c). Finally, the temporal evolution of the mean speed shows an initial average velocity of approximately 0.3 μm/s that gradually decelerates to below 0.1 μm/s over the observation window (Figure. 3d).

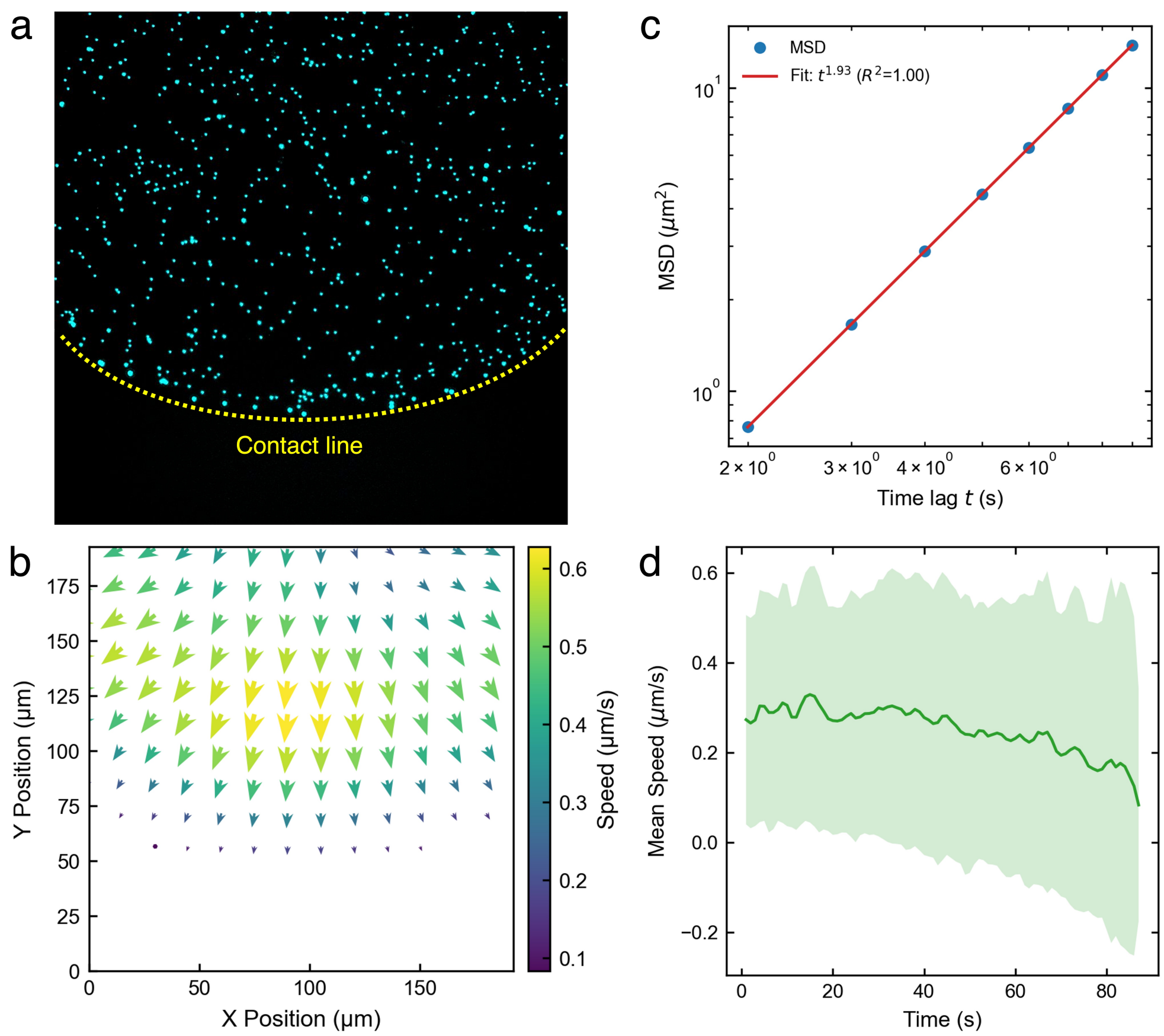

Figure S3: Evaporative dynamics of lattice quantum dots. (a) Microscopy image. (b) Velocity vector field revealing outward capillary flow. (c) Mean squared displacement (MSD) showing ballistic transport ($\alpha = 1.93$). (d) Temporal evolution of the mean speed over time.

Gel-like mechanical properties of the Pluronic film

To investigate the mechanical properties of the Pluronic film and its ability to incorporate larger particles, we performed experiments with 1 μm carboxylate-modified polystyrene beads embedded in the Pluronic solution (1% w/w in M2B buffer). These beads are significantly larger than the quantum dots (15–20 nm) and serve as mechanical probes to assess the material properties of the depositing polymer network. The experiments were visualized using both polarized light and fluorescence microscopy to simultaneously observe the bead dynamics and the evolving lattice pattern.

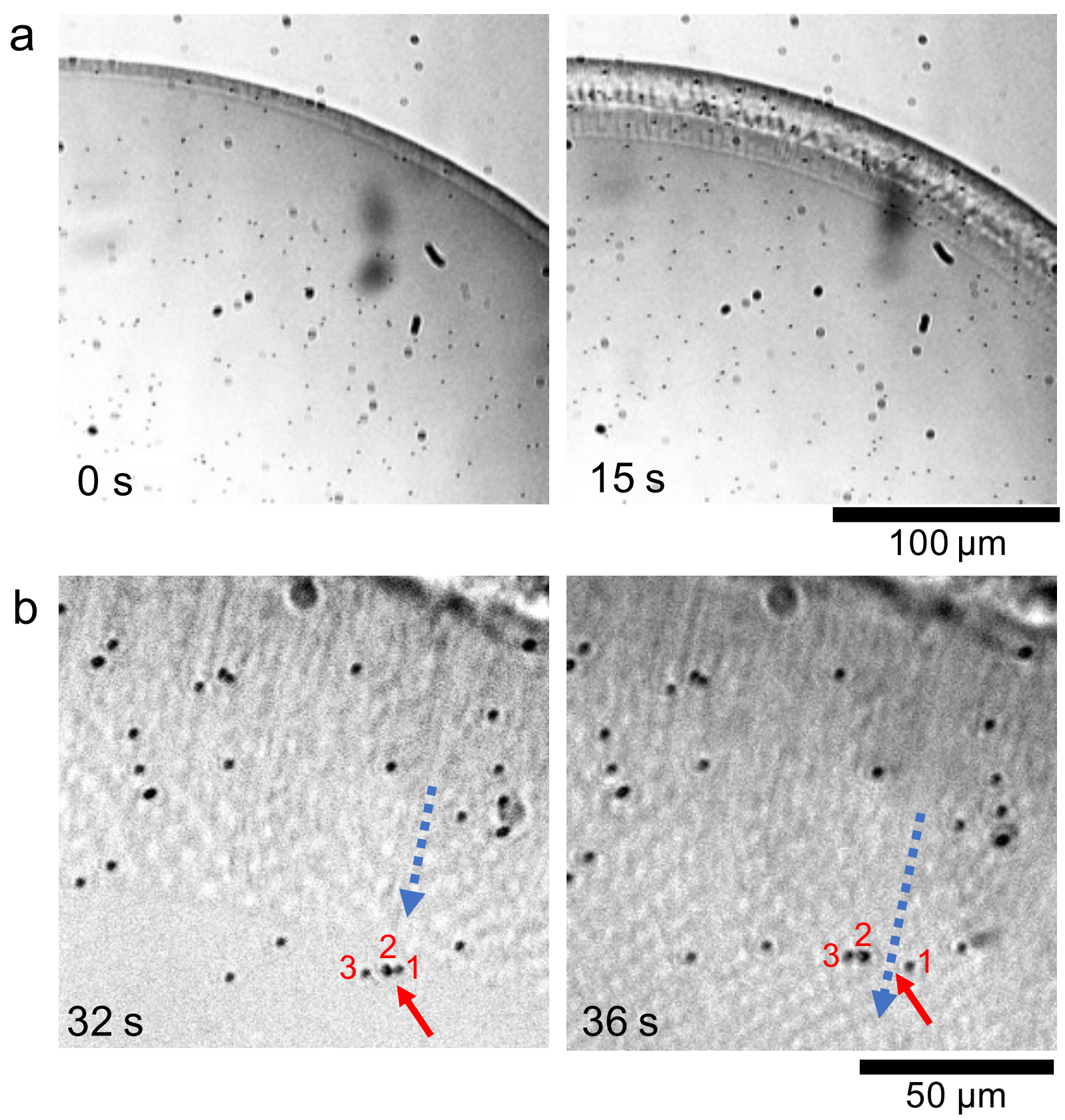

Figure S4: Dynamics of 1-μm polystyrene beads within the evaporating Pluronic droplet during lattice pattern formation. a) Polarized light microscopy snapshots showing droplet evolution and lattice emergence over time. b) Magnified view highlighting beads embedded within the lattice. The blue dashed line indicates a radial rip, demonstrating that beads can be laterally displaced by propagating defects. These observations suggest that the Pluronic film exhibits gel-like mechanical properties, capable of incorporating and displacing micron-sized particles.

Deposition dynamics of Pluronic F-127 in PBS

To evaluate how specific electrolyte composition influences interfacial pattern formation, control experiments were performed using 1% w/w Pluronic F-127 dissolved in standard phosphate-buffered saline (PBS) under identical drying conditions. While the presence of salt in PBS is sufficient to trigger contact-line instabilities due to moderate salting-out effects, the resulting morphology differs substantially from the complete buffer system. Evaporation produces radial, finger-like instabilities near the receding contact line; however, these instabilities fail to undergo the secondary lateral connectivity required to assemble into an ordered, highly symmetric lattice network. This control confirms that ionic strength and specific ion composition govern both the microphase separation threshold and the structural evolution of the deposited network.

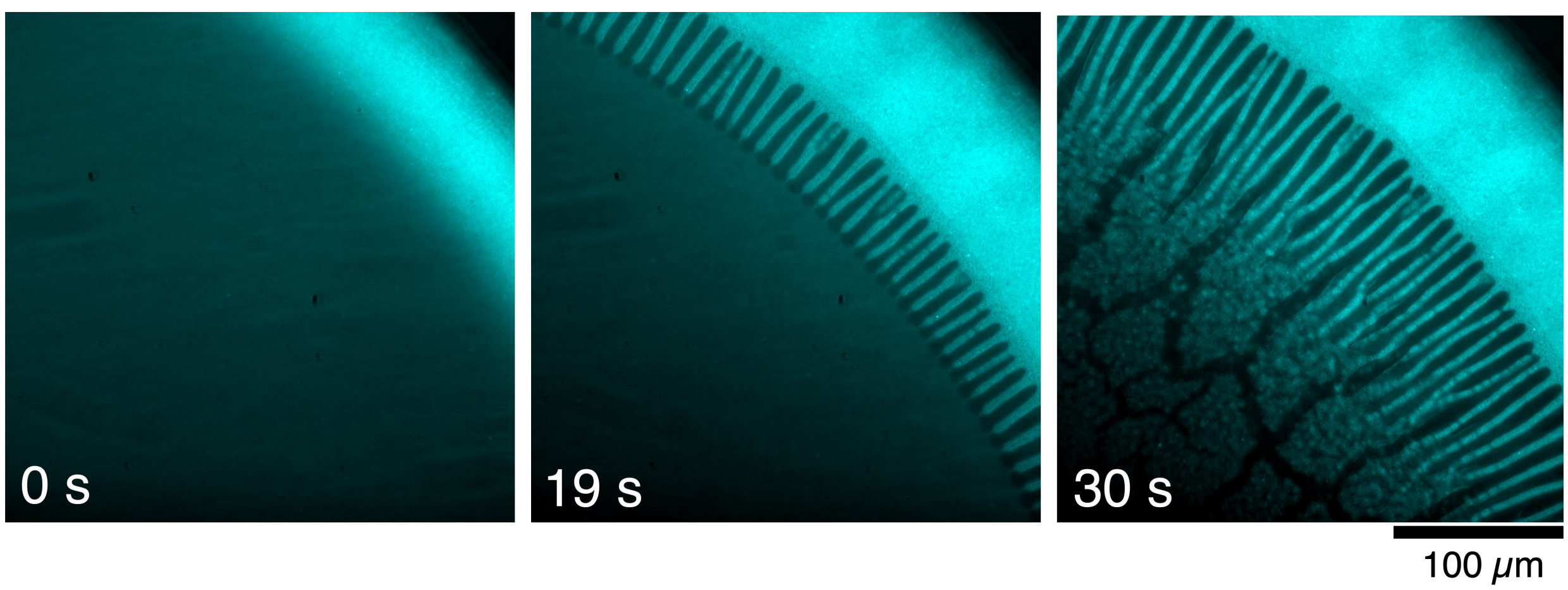

Figure S5: Evaporation and pattern formation of a 1% w/w Pluronic F-127 droplet dissolved in phosphate-buffered saline (PBS). Time-lapse fluorescence images showing the emergence of radial finger-like instabilities along the receding contact line.

Supplementary Videos

Video S1. Time-lapse fluorescence microscopy of an evaporating sessile droplet containing 0.5% w/w Pluronic F-127 in M2B buffer, showing the formation and merging of coacervate clusters at the receding contact line.

Video S2. Time-lapse fluorescence microscopy of an evaporating sessile droplet containing 1% w/w Pluronic F-127 in M2B buffer, showing the emergence of the regular lattice pattern and radial rips at the receding contact line.

Video S3. Time-lapse microscopy of the lattice pattern formation in a 1% w/w Pluronic droplet, highlighting the propagation of radial rips through the lattice sheet and the resulting crack-like defects that do not disrupt the overall lattice structure.

Video S4. Side-by-side view of the evaporating droplet. The left panel shows polarized light microscopy revealing the lattice pattern, and the right panel displays fluorescent imaging of the 2 μm beads used to track the internal flow of the droplet throughout the evaporation.

TOC Graphic

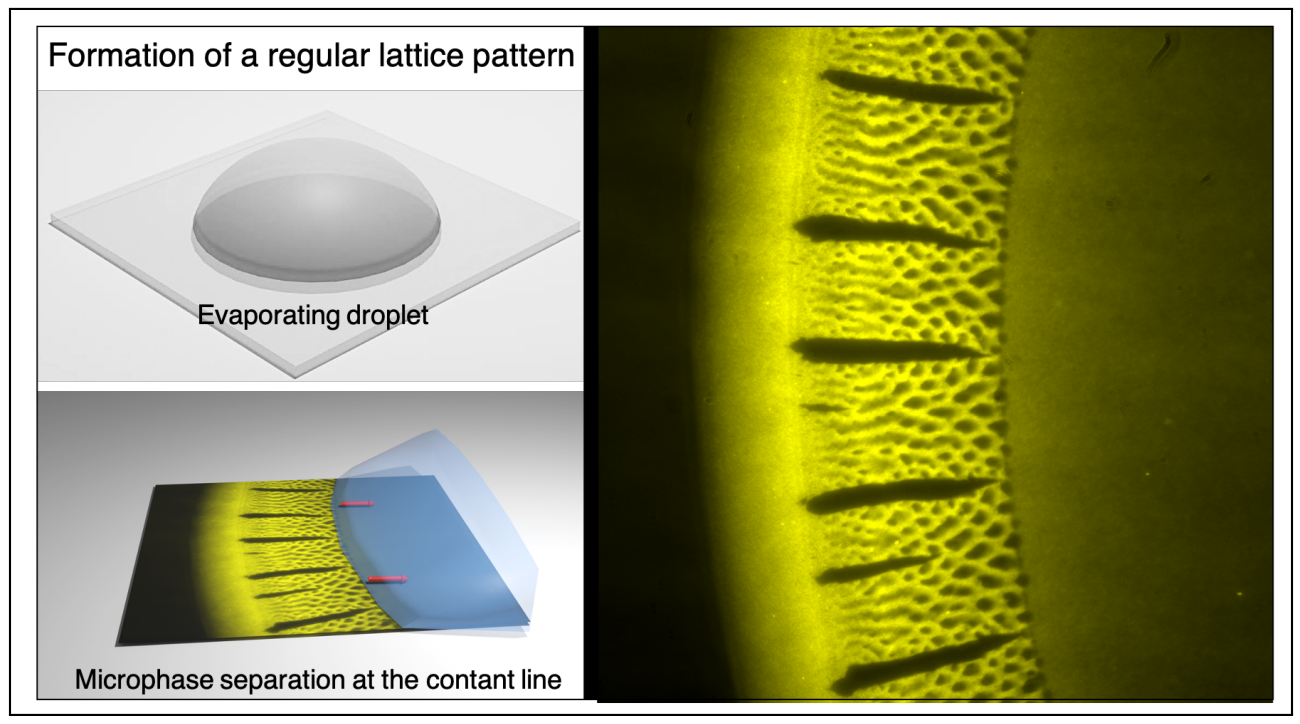
Formation of a regular lattice pattern
Evaporating droplet
Microphase separation at the contant line
